# Rosemary Extract-Induced Autophagy and Decrease in Accumulation of Collagen Type I in Osteogenesis Imperfecta Skin Fibroblasts

**DOI:** 10.3390/ijms231810341

**Published:** 2022-09-07

**Authors:** Joanna Sutkowska-Skolimowska, Justyna Brańska-Januszewska, Jakub W. Strawa, Halina Ostrowska, Malwina Botor, Katarzyna Gawron, Anna Galicka

**Affiliations:** 1 Department of Medical Chemistry, Medical University of Bialystok, Mickiewicza 2A, 15-222 Bialystok, Poland; 2 Department of Biology, Medical University of Bialystok, Mickiewicza 2A, 15-222 Bialystok, Poland; 3 Department of Pharmacognosy, Medical University of Bialystok, Mickiewicza 2A, 15-230 Bialystok, Poland; 4 Department of Molecular Biology and Genetics, Faculty of Medical Sciences in Katowice, Medical University of Silesia, Medykow 18, 40-475 Katowice, Poland

**Keywords:** rosemary extract, collagen type I, unfolded protein response, autophagy, proteasome, skin fibroblasts, apoptosis, osteogenesis imperfecta

## Abstract

Osteogenesis imperfecta (OI) is a heterogeneous connective tissue disease mainly caused by structural mutations in type I collagen. Mutant collagen accumulates intracellularly, causing cellular stress that has recently been shown to be phenotype-related. Therefore, the aim of the study was to search for potential drugs reducing collagen accumulation and improving OI fibroblast homeostasis. We found that rosemary extract (RE), which is of great interest to researchers due to its high therapeutic potential, at concentrations of 50 and 100 µg/mL significantly reduced the level of accumulated collagen in the fibroblasts of four patients with severe and lethal OI. The decrease in collagen accumulation was associated with RE-induced autophagy as was evidenced by an increase in the LC3-II/LC3-I ratio, a decrease in p62, and co-localization of type I collagen with LC3-II and LAMP2A by confocal microscopy. The unfolded protein response, activated in three of the four tested cells, and the level of pro-apoptotic markers (Bax, CHOP and cleaved caspase 3) were attenuated by RE. In addition, the role of RE-modulated proteasome in the degradation of unfolded procollagen chains was investigated. This study provides new insight into the beneficial effects of RE that may have some implications in OI therapy targeting cellular stress.

## 1. Introduction

Osteogenesis imperfecta (OI) is a rare hereditary bone disease, with a frequency of 1 in 15,000 to 20,000 live births, characterized by phenotypic and genotypic heterogeneity [1,2]. The most common symptoms include bone fragility, skeletal deformities, reduced bone mineral density, short stature, blue sclera, dentinogenesis imperfecta, hearing impairment, joint hypermobility, skin fragility, muscle weakness, and cardio-respiratory defects [1,2,3].

Despite recent discoveries of mutations in many genes, the most common are mutations in genes encoding type I collagen, which cause the majority (85%) of cases of OI [1,2,3,4,5]. Mutations in the *COL1A1* or *COL1A2* genes encoding the α1(I) and α2(I) chains of type I collagen, respectively, are dominant and cause quantitative or structural disturbances in collagen. The classification of OI due to collagen mutations includes four types, the phenotype of which ranges from mild (type I) and moderate (type IV) to severe (type III) and perinatal lethal in OI type II [6]. The molecular defect in OI type I is a null *COL1A1* allele due to premature stop codons, either directly or through frame shifts resulting in reduced synthesis of functional collagen type I [4,5,7]. OI types II-IV are mainly caused by substitutions of glycine residues by another amino acid (80%), but also by splicing site mutations as well as small triplet deletion or duplication mutations, which shift the register of α-chains in the helix [2,4,5,8]. Most of them result in the synthesis of mutant misfolded collagen molecules. In recent years, many other causative genes associated with recessive and X-linked forms of the disease have been detected. Most of these genes code for type I collagen-related proteins that play an important role in folding and post-translational modifications, secretion as well as quality control of collagen synthesis. Mutations of proteins unrelated to collagen type I play an important role in osteoblast maturation and bone mineralization [2,3,5,8,9,10].

Synthesis of type I collagen is a complex process including the intracellular and extracellular steps preceding the formation of mature collagen fibrils. Two proα1 and one proα2 chain are synthesized in the endoplasmic reticulum (ER) and undergo important post-translational modifications prior to triple helix folding, including hydroxylation of proline (at C-4 and C-3) and lysine residues [11,12], which have fundamental importance for the stability of the helix. Proper folding and post-translational modifications in the ER determine the effectiveness of collagen secretion to the extracellular matrix (ECM) [11,12]. Glycine substitutions are responsible for the delay in the formation of the triple helix. Prolonged exposure of procollagen chains to post-translational modifying enzymes leads to increased hydroxylation of proline and lysine residues and glycosylation of hydroxylysine residues, causing the synthesis of collagen molecules with the abnormal structure [2,4,5,8,13]. The mutated collagen is secreted into ECM, but it may be partially retained in the ER causing cellular stress, which may be related to the clinical outcome [14,15,16,17,18]. It has been reported that some OI cells activate an unfolded protein response (UPR) to restore cell homeostasis [14,17,18,19,20,21]. The best-studied three ER membrane receptors of UPR include inositol requiring enzyme 1 (IRE1), PKR-like endoplasmic reticulum kinase (PERK), and activating transcription factor 6 (ATF6) [19,20,22]. Under normal conditions, the chaperone—binding immunoglobulin protein (BiP) binds all three sensor proteins in their ER luminal domain and keeps them inactive, while under stressful conditions it binds preferentially to misfolded proteins leading to activation of UPR pathways. ATF6 moves to the Golgi apparatus, where it is cleaved by different proteases and then, as an active transcription factor, enters the nucleus and activates the promoter of its related target genes. IRE1 and PERK are activated by autophosphorylation and oligomerization. Activated IRE1 forms an alternative spliced variant of the X-Box binding protein 1 (XBP1s), which, as a transcription factor, increases the expression of various chaperones and proteins involved in the proteasomal of ER-associated degradation (ERAD). The activation of PERK inhibits global protein synthesis through phosphorylation of eukaryotic translation initiation factor (eIF2α) but favors the translation of some mRNAs, such as the activating transcription factor 4 (ATF4), which is involved in both cell survival and ER stress-dependent apoptosis. During chronic stress, ER promotes apoptosis by upregulating genes such as the homologous protein of the CCAAT enhancer binding protein (CHOP) [20,22,23]. If conformation of mutated collagen is not improved by chaperones, it is destined for degradation most often via autophagy [24]. 

Autophagy is a complex system regulated by more than 30 autophagy-related gene (ATG) proteins. The most studied markers associated with autophagy are beclin 1, a microtubule-associated protein 1 light chain 3 (LC3), and sequestosome 1 (SQSTM1/p62), later referred to as p62 [25]. In some cases, especially the mutations occurring in C-propeptide, that most affect the trimer assembly, the retrotranslocation of misfolded procollagen chains into the cytosol may occur and result in their degradation by the proteasome [26,27]. 

So far, anti-catabolic bisphosphonates, denosumab as a synthetic parathyroid hormone and growth hormone have been used in the therapy of OI [1,3,10]. Experimental OI therapy strategies such as genetically engineered stem cell transplantation methods, reprogramming of somatic cells into pluripotent stem cells as well as anti-transforming growth factor (TGF-β) therapy have been summarized by us recently [28]. The major disadvantages of these therapies are their poor efficacy, or cytotoxic side effects. Research is still underway to find new and more effective drugs. According to the latest research, the chemical 4-phenylbutyrate chaperone (4-PBA) is of great interest, the molecular target of which is ER stress caused by intracellular retention of mutant collagen in osteoblasts and fibroblasts [16,17,18,29,30]. 

Medicinal plants containing significant amounts of polyphenols are of great interest to researchers due to their high therapeutic potential. Polyphenols are widely studied as regulators of fundamental biological processes, including cell proliferation, apoptosis, and autophagy [31,32]. Rosemary (*Rosmarinus officinalis* L., Lamiaceae) is a rich source of many bioactive polyphenol compounds with a wide range of biological activities, such as antioxidant, antimicrobial, anti-inflammatory, antidiabetic and anticancer [33,34]. In our previous study, we showed a beneficial effect of rosemary extract (RE) on the biosynthesis of type I collagen in OI type I fibroblasts with quantitative type I collagen defect [35]. This time we examined whether RE could reduce the accumulation of mutant collagen in the fibroblasts of two patients with severe type III and two patients with lethal type II OI carrying mutations in α(I) chain. In addition, possible mechanisms involved in the action of RE were also investigated. 

## 2. Results

### 2.1. Steady-State Collagen Analysis in OI Fibroblasts

**Figure 1:**
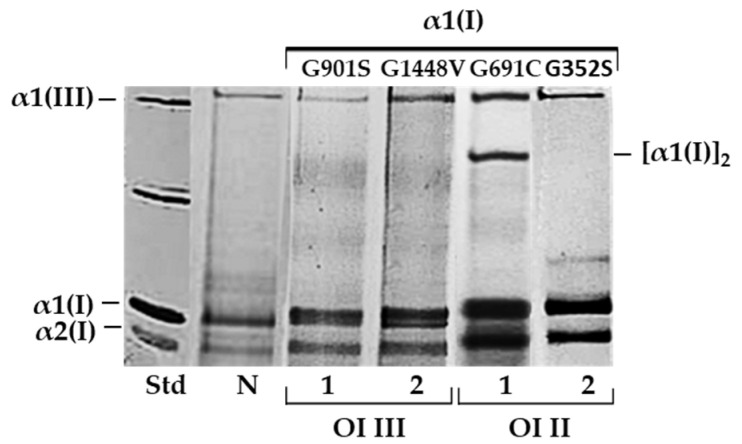
SDS-urea polyacrylamide gel electrophoresis (SDS-urea PAGE), in non-reducing conditions, of collagen type I in fibroblasts of patients 1 and 2 OI types III and II with glycine substitutions in α1(I) chain. To detect collagen after electrophoretic separation, silver staining was used; N—normal cells, Std—standard (bovine collagen type I) (Biocolor Life Science, UK).

To identify mutated collagen, the mobility of the α1(I) and α2(I) bands was analyzed by SDS-urea polyacrylamide gel electrophoresis (SDS-urea PAGE) (Figure 1). All cells with glycine substitution (G910S and G1448V in patients 1 and 2 with severe type III, and G691C and G352S in patients 1 and 2 with lethal type II OI) in the α1(I) showed delayed migration of α1(I) and α2(I) chains and intracellular retention. In OI type II (patient 1) cells, an additional band [α1(I)]_2_ dimer was identified between the α1(I) monomers and the α1(III) trimer, confirming the substitution of glycine with cysteine (G691C).

### 2.2. Reduced Accumulation of Collagen Type I in OI Fibroblasts Treated with Rosemary Extract 

**Figure 2:**
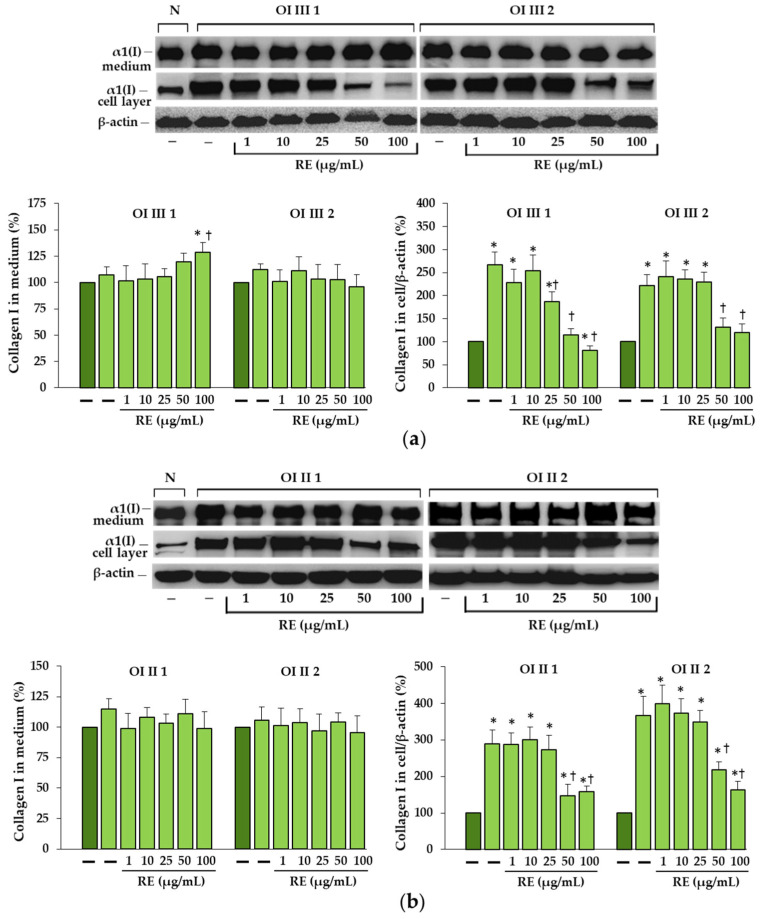
The effect of rosemary extract (RE) on the levels of secreted (in medium) and intracellularly accumulated type I collagen in OI type III (**a**), and OI type II (**b**) cells; β-actin was used as cell protein loading control. The bars represent the results of the gel densitometry as the mean values from three independent experiments; * *p* < 0.05, OI cells vs. normal (N) cells; ^†^ *p* < 0.05, OI treated cells vs. OI untreated cells. The data are expressed as a percentage of the normal sample taken as 100%; dark green and light green bars represent normal and OI, respectively.

In untreated OI fibroblasts, the accumulation of collagen type I in all four cell lines was confirmed by Western blot (Figure 2). When OI cells were treated with RE at the concentrations of 1–100 µg/mL, the level of accumulated collagen in cell layers was significantly reduced at 25, 50 and 100 µg/mL RE in OI type III patient 1, and at the concentrations of 50 and 100 µg/mL RE in the rest of the cells. Moreover, in cells of OI III 1 (at 50 µg/mL RE) and OI III 2 (at 50 and 100 µg/mL RE) the level of collagen type I was normalized (Figure 2a). The level of collagen secreted by OI fibroblasts, apart from the increase in the medium of OI III 1 fibroblasts treated with 100 µg/mL RE, remained unchanged. It should be added that RE at these concentrations did not significantly affect the viability of OI cells (Supplementary Figure S1).

Since rosmarinic acid (RA) is an essential component of RE, we also examined its effect on the mutant collagen retention. RA in a wide range of concentrations (1–100 µM), apart from lowering the accumulation of collagen in OI II 1 cells at its highest concentration (100 µM), had no effect in the remaining cells (Supplementary Figure S2). In the subsequent studies, we focused on the mechanisms of action of RE at the concentrations of 50 and 100 µg/mL.

### 2.3. Activation of Unfolded Protein Response in OI Cells

**Figure 3:**
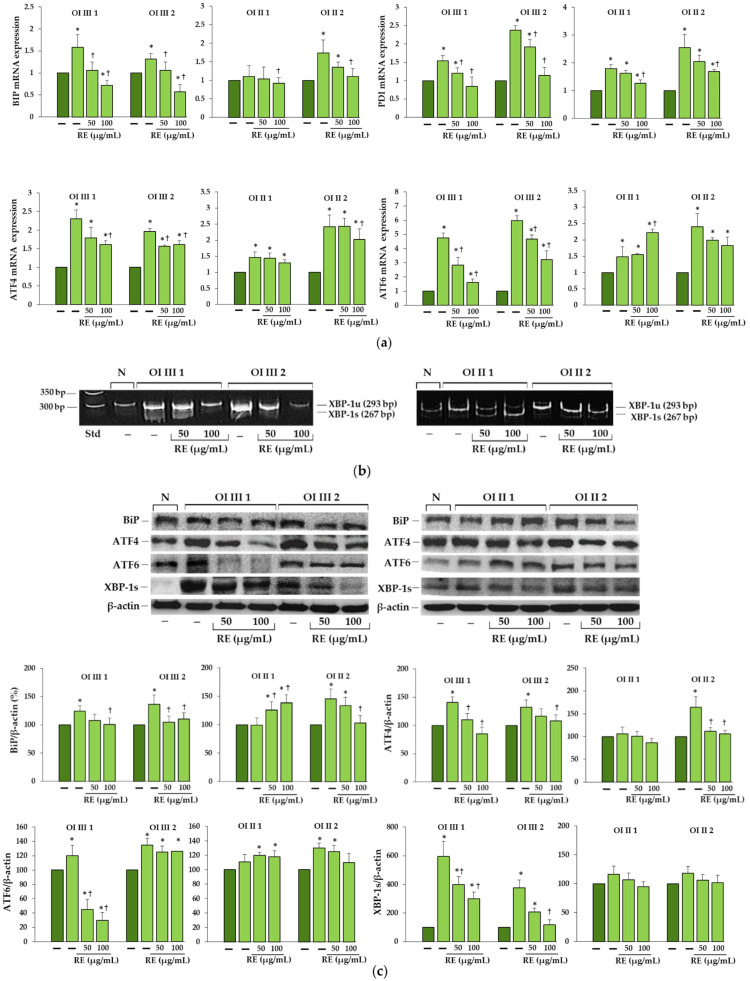
(**a**) The influence of rosemary extract (RE) on mRNA relative expression of binding immunoglobulin protein (BiP), protein disulfide isomerase (PDI), and activating transcription factors 4 and 6 (ATF4, ATF6) in OI types III and II cells. The bars represent the results of the mean values from three independent experiments; * *p* < 0.05, OI cells vs. normal cells; ^†^ *p* < 0.05, OI treated cells vs. OI untreated cells; dark green and light green bars represent normal and OI, respectively. (**b**) Unspliced and spliced X-box binding protein 1 (Xbp-1u and Xbp-1s) RT-PCR products were analyzed on 7% polyacrylamide gel. (**c**) Western blot analysis of BiP, ATF4, ATF6 and XBP-1s in OI type III and II cells; β-actin was used as cell protein loading control. The bars represent the results as the mean values from three independent experiments; * *p* < 0.05, OI cells vs. normal (N) cells; ^†^ *p* < 0.05, OI treated cells vs. OI untreated cells. The data are expressed as a percentage of the normal sample taken as 100%; dark green and light green bars represent normal and OI, respectively.

To determine whether retained mutant procollagen activates UPR, the expression of BiP, protein disulfide isomerase (PDI), ATF4, AFT6 and XBP-1s was evaluated (Figure 3).

Expression of the chaperone BiP, which is an activator of UPR sensors, was significantly increased in untreated OI cells at the mRNA level beyond OI II 1 cells where it remained unchanged compared to the normal cells (Figure 3a). PDI, which catalyzes the formation and isomerization of disulfide bonds and acts as a collagen chaperone, was significantly increased in all untreated OI cells at mRNA level (Figure 3a). In the presence of RE (50 and 100 µg/mL), the expression of both chaperone transcripts was mostly reduced to a different extent compared to the untreated OI cells (Figure 3a).

ATF4, which is the effector in the PERK branch, was according to the analysis of its transcript expression, upregulated in untreated OI cells. RE either did not change or it decreased the expression of this factor mRNA, dependently on RE concentration, in comparison to the untreated OI cells (Figure 3a).

Expression of ATF6 was also investigated, and the level of its mRNA in untreated OI cells increased compared to the control. In cells exposed to RE (50 and 100 µg/mL) the decrease in ATF6 gene expression was noted in OI III, no effect in OI II 2 or stimulating effect of 100 µg/mL RE in OI II 1 (Figure 3a). 

Activation of the IRE1α branch was studied by determining the level of XBP-1 expression in which splicing is mediated by this protein. In normal cells, the splicing form of XBP-1 (XBP-1s) was absent, while in OI it was manifested mainly in type III OI and was barely detectable in OI II cells (Figure 3b). Under the influence of RE at a concentration of 100 µg/mL, the spliced form disappeared in OI type III and only the unspliced (XBP-1u) form was observed, while in RE-treated OI II cells a slower migrating band appeared, with greater intensity in OI II 1 (Figure 3b). To find out whether the changes in the expression of proteins included in the UPR in OI cells reflect the expression of their genes, Western blots were performed (Figure 3c).

The results of the Western blot analysis revealed a similar increase in BIP at the protein level as was at the mRNA level, and no increase in untreated OI II 1 cells where no upregulation of the BIP gene was shown. Interestingly, RE at both concentrations (50 and 100 µg/mL) increased BIP expression in OI II 1 cells, and in others with upregulation of this chaperone protein, the RE induced decrease in relation to untreated OI cells was noted (Figure 3c). The expression of ATF4 and ATF6 transcription factors at the protein level was increased as at the mRNA level, except for OI II 1 cells, where their genes were upregulated. In RE-treated cells, the normalization of ATF4 level was achieved, while the ATF-6 level was either unaffected or significantly decreased by RE in OI III 1 cells (Figure 3c). XBP-1s at the protein level was almost undetectable in normal age-matched cells for OI type III, while its expression increased almost 6-fold and 4-fold in OI III 1 and 2 cells, respectively, and significantly decreased under the influence of RE. In contrast, in untreated OI II cells, only an upward trend and no RE effect on the expression of this spliced form of the factor was observed (Figure 3c).

### 2.4. Rosemary Extract-Induced Autophagy in OI Cells 

**Figure 4:**
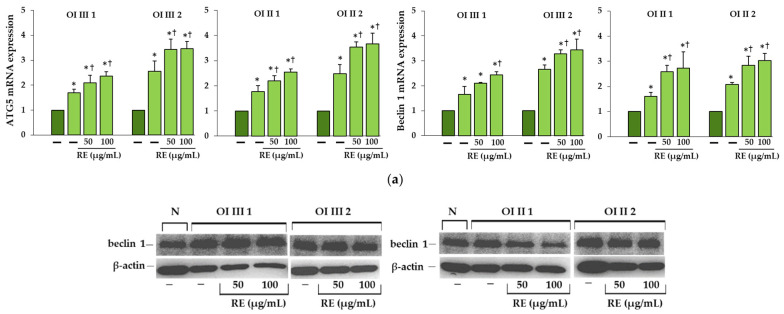

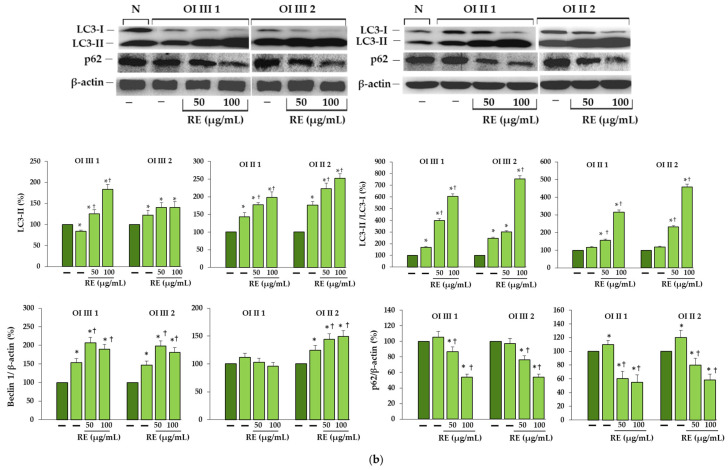
(**a**) The influence of rosemary extract (RE) on mRNA relative expression of autophagy-related gene (ATG5) and beclin 1 in OI types III and II cells. The bars represent the results of the mean values from three independent experiments; * *p* < 0.05, OI cells vs. normal cells; ^†^ *p* < 0.05, OI treated cells vs. OI untreated cells; dark green and light green bars represent normal and OI, respectively. (**b**) Western blot analysis of beclin 1, microtubule-associated protein 1 light chain 3 (LC3-I/LC3-II), and sequestosome 1 (SQSTM1/p62) in OI type III and II cells; β-actin was used as cell protein loading control. The bars represent the results as the mean values from three independent experiments; * *p* < 0.05, OI cells vs. normal (N) cells; ^†^ *p* < 0.05, OI treated cells vs. OI untreated cells. The data are expressed as a percentage of the normal sample taken as 100%; dark green and light green bars represent normal and OI, respectively.

ATG5 and beclin 1, which are important in initiation of autophagosome formation, increased in all untreated OI cells at the mRNA level and their expression intensified in the presence of RE (Figure 4a). In addition, the expression levels of autophagy related markers were determined by Western blot as well. Beclin 1 level increased in untreated OI III and OI II 2 and even more in RE treated cells consistent with gene expression, while there was no change in untreated and RE treated OI II 1 cells compared to normal cells (Figure 4b). LC3-II as a marker of the final fusion of the autophagosome with the lysosome was increased in untreated OI cells apart for OI III 1. In the presence of RE (50 and 100 µg/mL), no effect in OI III 2 and significant stimulation of LC3-II expression in the remaining cells was demonstrated as compared to untreated cells (Figure 4b).

In order to assess the dynamic process of autophagy, the ratio of LC3-II to LC3-I was determined, which in untreated cells either increased (in OI III 1 and 2) or remained unchanged (in OI II 1 and 2) compared to normal, while RE significantly induced autophagic flow in all OI cells (Figure 4b). The expression of p62, which is an indicator of autophagic degradation, was unchanged in OI III and increased in OI II cells. We examined whether RE facilitates degradation of p62. Treatment of cells with RE significantly decreased the p62 level, which suggests RE-induced autophagic degradation (Figure 4b). Since p62 and LC3-II are also degraded along with the digestion of cell components by autophagy; therefore, in order to further confirm that the increase in LC3-II induced by RE was not indicative of accumulation of this protein, ammonium chloride (NH_4_Cl) as a lysosome proteolysis inhibitor was used. In all OI cells with inhibited lysosomal protein degradation, not only the LC3-II level but also p62 were significantly higher than in cells treated with RE alone (Supplementary Figure S3). 

**Figure 5:**
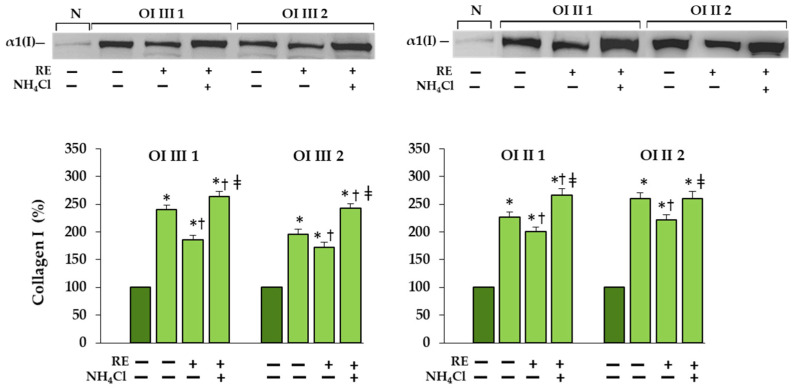
Western blot analysis of type I collagen in the lysosome-enriched cellular fraction, prepared as described in Methods, in OI types III and II cells treated with 50 µg/mL RE and 50 mM NH_4_Cl. Equal amounts of proteins were loaded on 10% polyacrylamide gel. The bars represent the results of the gel densitometry as the mean values from three independent experiments; * *p* < 0.05, OI cells vs. normal (N) cells; ^†^ *p* < 0.05, OI cells treated with RE vs. OI untreated cells; ^‡^ *p* < 0.05, OI cells treated with RE + NH_4_Cl vs. OI cells treated with RE alone. The data are expressed as a percentage of the normal sample taken as 100%; dark green and light green bars represent normal and OI, respectively.

To confirm that collagen type I is degraded in the lysosomal pathway, a lysosome-enriched cell fraction was prepared and tested for its level in the presence and absence of lysosomal inhibitor in RE treated cells. We found that collagen type I was localized to a very small extent in the lysosomal fraction of normal cells and to a much greater extent in untreated OI cells (Figure 5). In OI cells treated with RE alone the level of type I collagen was lower than in the presence of inhibitor of lysosomes NH_4_Cl, which indicates its lysosomal degradation.

**Figure 6:**
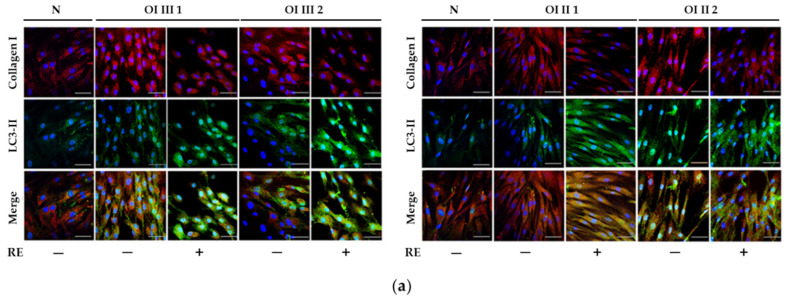

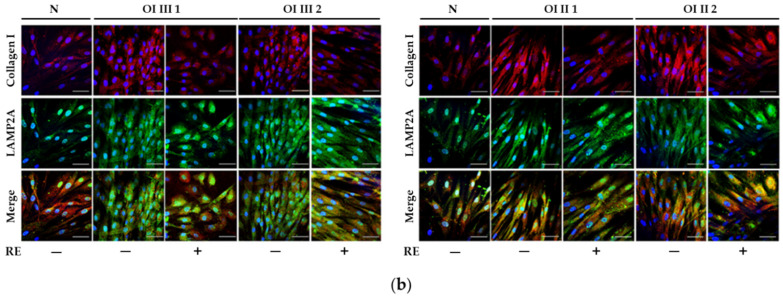
Colocalization of collagen type I (red)/LC3-II (green) (**a**), and of collagen type I (red)/LAMP2 (green) (**b**), and changes in the intensity of staining of these proteins in normal (N) and OI types III and II cells, treated with 50 µg/mL rosemary extract (RE), revealed by confocal microscopy analysis. A merge signals of colocalizing collagen type I/LC3-II and collagen type I (/LAMP2 were detectable, with greater intensity in RE-treated cells. Nuclei were stained with DAPI (blue). Scale bar, 50 μm. Representative immunofluorescent images are shown.

Using confocal fluorescence microscopy with immunofluorescence staining, representative images of which are presented in Figure 6, we assessed the localization of endogenous collagen I, LC3-II and lysosomal-associated membrane protein 2A (LAMP2A) in normal and OI cells. It was observed that compared to normal cells, collagen type I staining intensity (red) in untreated OI cells increased, and after treatment with RE it decreased, whereas LC3-II (green) inversely decreased in untreated OI cells and increased in the presence of RE. Similar to the Western blot results, collagen type I staining decreased in the presence RE; moreover, it was co-localized with LC3-II as evidenced by the merged images (Figure 6a). We additionally assessed co-localization of collagen type I and LAMP2A, which is a lysosomal marker. As shown in Figure 6b, co-immunofluorescence staining for collagen type I (red) and LAMP2A (green) showed the increase in their co-localization after treatment of OI cells with RE. The above results suggest a stimulating effect of RE on the intracellular collagen degradation mediated by the autophagolysosomes.

### 2.5. Involvement of Proteasome in Degradation of Unfolded Procollagen Chains in OI III 2 Cells 

**Figure 7:**
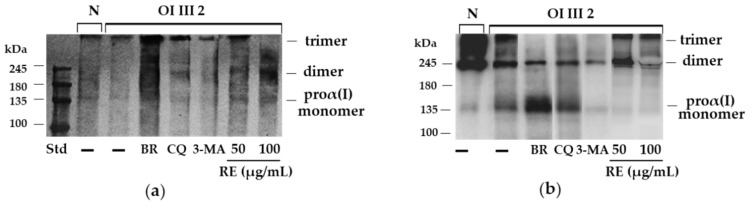
Polyubiquitynation (**a**) and SDS-PAGE of silver-stained (**b**) procollagen type I monomers, dimers and trimers, performed under non-reducing conditions in normal (N) and OI III 2 cells treated with 50 and 100 µg/mL rosemary extract (RE), 50 nM bortezomib (BR), 50 µM chloroquine (CQ), and 5 mM 3-methyladenine (3-MA).

Since it has been reported that unfolded procollagen chains can be degraded by the ERAD pathway [27], we checked the polyubiquinination of type I procollagen in OI cells following SDS-PAGE under non-reducing conditions. As it turned out, under these conditions, polyubiquitinated proteins corresponding to the molecular weight of procollagen chains were detected only in OI III 2 cells, and the increase in this modification was noted in the presence of RE (Supplementary Figure S4). In order to determine the way of their degradation, a Western blot of polyubiquitinated proteins (Figure 7a) and SDS-PAGE of silver-stained proteins (Figure 7b) in the presence of proteasome and autophagy inhibitors were performed. Three polyubiquitinated protein bands corresponded to monomers, dimers and trimers of type I procollagen. Dimers and trimers are stabilized by the formation of inter-chain disulfide bonds within the C-propeptide. There was the increased level of unfolded proα1(I) chains and a decreased level of trimers in OI cells compared to the control cells (Figure 7b), while the level of monomer polyubiquitination was slightly reduced in OI cells (Figure 7a). In the presence of bortezomib (BR), a proteasome inhibitor, increased levels of polyubiquitinated monomers correlated with increased level of unfolded procollagen chains. Treatment with autophagy inhibitors chloroquine (CQ) and 3-methyladenine (3-MA) remained without effect on the level of polyubiquitination, but interestingly, the level of unfolded procollagen chains in the presence of CQ slightly increased and under the influence of 3-MA decreased as compared to untreated OI cells. Rosemary extract caused the increase in the polyubiquitination of monomers and dimers (Figure 7a), which was accompanied by a marked decrease in the level of unfolded collagen (Figure 7b).

**Figure 8:**
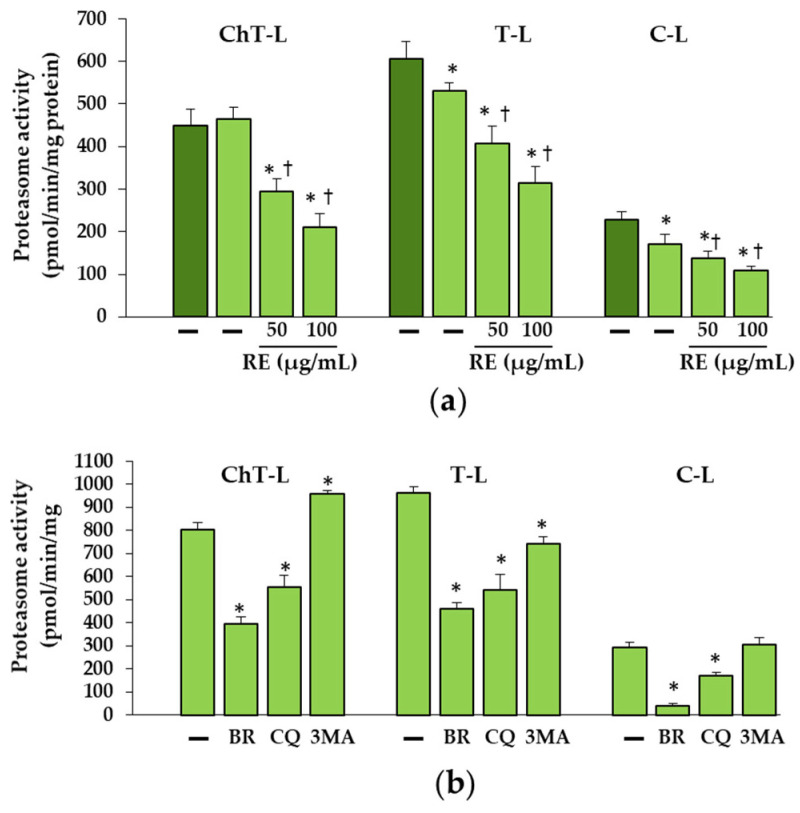
Proteasomal activities: chymotrypsin-like (ChT-L), trypsin-like (T-L) and caspase-like (C-L) in OI III 2 cells treated with 50 and 100 µg/mL rosemary extract (RE) (**a**), 5 nM bortezomib (BR), 50 µM chloroquine (CQ) and 5 mM 3-methyladenine (3-MA) (**b**). The bars represent the mean values from three independent experiments. (**a**) * *p* < 0.05, OI cells vs. normal cells; ^†^ *p* < 0.05, OI cells treated with RE vs. OI untreated cells; dark green and light green bars represent normal and OI, respectively; (**b**) * *p* < 0.05, OI cells treated with inhibitors vs. OI untreated cells.

Additionally, the effect of RE on proteasomal activities: chymotrypsin-like (Ch-L), trypsin-like (T-L) and caspase-like (C-L) in untreated and RE-treated OI fibroblasts was examined. As shown in Figure 8a, the decrease in T-L and C-L activities and no change in ChT-L activity were found in untreated OI cells, while in the presence of RE all activities were lowered as compared to untreated cells. The effectiveness of BR was confirmed by inhibitory effect on all proteasome activities (Figure 8b). The use of autophagy inhibitors, in turn, resulted in a slight stimulation of ChT-L activity by 3-MA and an inhibitory effect of CQ on all proteasome activities (Figure 8b). 

To check whether the inhibition of proteasome activity by RE does not lead to the accumulation of non-collagen proteins, the level of silver-stained lysate proteins separated on 10% gels under reducing conditions was assessed and additionally the level of polyubiquitination of these proteins (Supplementary Figure S5). The increase in the level of polyubiquitinated total proteins (Supplementary Figure S5a) coincided with the increase in the level of total protein expression in OI untreated cells (Supplementary Figure S5b) compared to the normal, which can be explained by the disruption of the autophagy and proteasome degradation processes. Treatment of OI cells with RE did not lead to the total protein accumulation, as was the case with the use of proteasome (BR and MG132) or autophagy (CQ, 3-MA and NH_4_Cl) inhibitors, their levels were comparable to the normal (Supplementary Figure S5b).

### 2.6. Expression and Activity of Collagen Type I Degrading MMPs 

Additionally, we assessed the expression of matrix metalloproteinases MMP-1 and MMP-2, degrading extracellular collagen I, in untreated and RE exposed OI cells. MMP-1 mRNA in OI cells was significantly upregulated compared to normal cells, while RE significantly decreased it. MMP-2 was upregulated at the mRNA level in OI type II and was normalized in the presence of both RE concentrations (50 and 100 µg/mL) in OI II 1 and at 100 µg/mL in OI II 2 (Supplementary Figure S6a).

The zymography allowed to identify mainly pro-MMP-2 in media of normal cells and additionally its active form MMP-2 in OI, while RE, beyond OI II 1, at a concentration of 100 µg/mL, showed a lowering effect, the highest in OI III 1 (Supplementary Figure S6b). 

### 2.7. Rosemary Extract Reduces the Expression of Apoptosis Markers in OI Cells 

**Figure 9:**
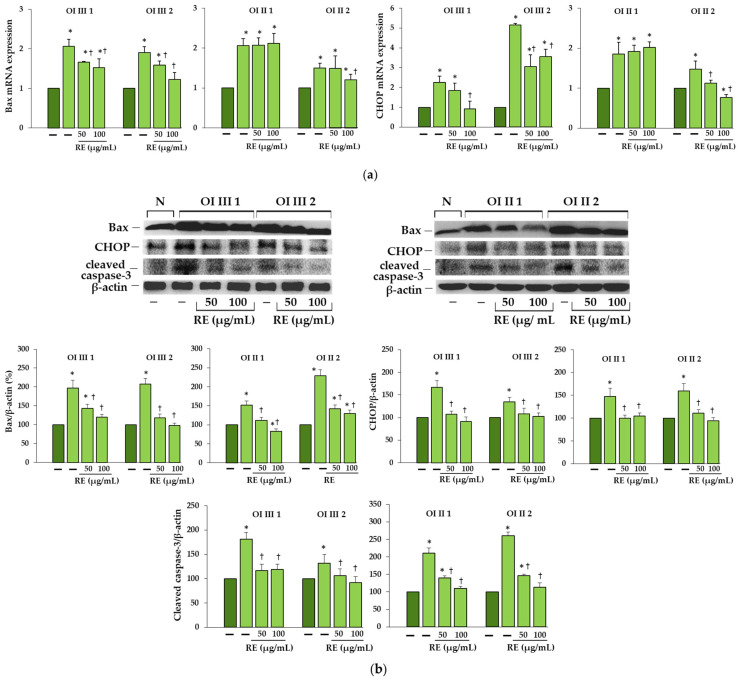
(**a**) The effect of rosemary extract (RE) on mRNA relative expression of Bax and CCAAT enhancer binding protein (CHOP) in OI types III and II cells. The bars represent the results of the mean values from three independent experiments; * *p* < 0.05, OI cells vs. normal cells; ^†^ *p* < 0.05, OI treated cells vs. OI untreated cells; dark green and light green bars represent normal and OI, respectively. (**b**) Western blot analysis of Bax, CHOP and cleaved caspase-3 in OI type III and II cells; β-actin was used as cell protein loading control. The bars represent the results as the mean values from three independent experiments; * *p* < 0.05, OI cells vs. normal (N) cells; ^†^ *p* < 0.05, OI treated cells vs. OI untreated cells. The data are expressed as a percentage of the normal sample taken as 100%; dark green and light green bars represent normal and OI, respectively.

The results presented in Figure 9 showed the significant increase in the expression of proapoptotic proteins such as Bax and CHOP at both mRNA and the protein levels as well as cleaved caspase-3 protein. Under the influence of RE at both concentrations (50 and 100 µg/mL), downregulation of these markers was mostly observed; even if the levels of Bax and CHOP mRNA did not change in OI II 1 cells, a significant decrease was observed at the protein level. 

## 3. Discussion

OI is a genetically and phenotypically heterogeneous group of connective tissue disorders caused mainly by an autosomal dominant mutations in collagen type I, which is the most abundant protein in bone and skin ECM [1,2,3,4,5,6,7,8,9,10]. The discovery in the last two decades of new causative genes (around twenty) that are involved in the regulation and function of type I collagen, but also in other aspects of bone biology [2,3,5,8,9,10] confirms the complexity of the underlying mechanisms and complicates understanding the relationship between mutations and phenotype as well as finding an effective and a universal method of treating the disease. Recently, ER stress caused by intracellular retention of mutant collagen in osteoblasts and fibroblasts was found as an attractive target of OI therapy [14,15,16,17,18,19,20,21]. The use of the chemical chaperone 4-PBA, approved by *Food and Drug Administration*, reduced ER stress and restored cell homeostasis in human fibroblasts of OI patients carrying dominant mutations in α1 and α2 collagen type I chains [17] as well as recessive mutations in cartilage-associated protein *(CRTAP*), prolyl-3-hydroxylase 1 (*P3H1*) and cyclophilin B (*PPIB*) impairing prolyl-3 hydroxylation of collagen type I [18]. Moreover, it was found that 4-PBA normalizes the overproduction of type I collagen and improves the misfolding of the type I collagen helix in OI fibroblasts due to glycine substitution, and also improves the impaired mineralization of osteoblasts differentiated from OI induced pluripotent stem cells [30]. Administration of this drug to the OI dominant zebrafish model, carrying typical glycine substitution G574D in *COL1A1*, alleviated cellular stress and improved bone mineralization in larvae and skeletal deformity in adults. This was accompanied by the reduction of the ER cisternae size and promoting the secretion of collagen [29]. Similarly, in osteoblasts of two murine OI models carrying G349C mutation in *COL1A1* (Brtl mouse) and G610C in *COL1A2* (Amish mouse), 4-PBA prevented collagen type I accumulation through increased its secretion and reduction of aggregates in mutant cells [16]. In addition, increased collagen incorporation into the matrix and improved mineral deposition in osteoblasts was observed in both murine models, which convinces about the influence of ER stress on the phenotype. Therefore, as well as the discovery of the therapeutic potential of this chemical chaperone, finding of other safe compounds to reduce cellular stress and restore cell homeostasis may be a new strategy for treating this disease, or at least some OI cases.

This study provides, for the first time, evidence of the beneficial effects of rosemary extract on fibroblasts with mutations in the collagen triple helix resulting in intracellular collagen accumulation. Moreover, the presented results explain the likely mechanisms of reducing cellular stress, mainly by enhancing the degradation of mutant collagen through autophagy. For our study, we chose glycine substitutions in the α1(I) chains of two patients with severe OI type III (G901S and G1448V), and two with lethal type II (G352S and G691C). As we demonstrated by SDS-urea-PAGE, all cells showed delayed migration of α1 and α2 collagen chains and intracellular accumulation of mutant collagen.

Collagen type I is characterized by a unique right-handed triple helical structure, and each of three left-handed polyproline-like helices contains a repeated sequence (Gly-X-Y) in which X and Y are often proline and hydroxyproline [36]. The triple helical domain is flanked on both sides by N- and C-terminal propeptides. Procollagen folding takes place in the ER, after which the protein is transported to the Golgi apparatus and secreted, where the cleavage of propeptides takes place and mature collagen is formed. Folding is a very complex process involving many chaperones, such as BiP, PDI, prolyl 4-hydroxylase, various peptidyl-prolyl *cis*-*trans* isomerases, and heat shock protein 47, whose role is to provide stabilization of the structure and to protect aggregation of unfolded chains [11,12,37]. 

We found increased BiP expression in two OI type III (G901S and G1448V) and one OI type II (G352S) cells, and a consequent activation of UPR pathways, whereas in OI II 1 cells with G691C the expression of this chaperone remained unchanged compared to the normal cells. According to Besio et al. [17] increased BiP was detected in three out of five tested fibroblasts with glycine substitution in α1 and α2 chains. Expression of PDI, which catalyzes the formation and isomerization of disulfide bonds and acts as a collagen chaperone by interacting with collagen α single chains, was upregulated in all α1(I) mutant cells, while in the study of Besio et al. [17] in four out five cells with mutations in α1(I) and it remained unchanged in cells with mutations in α2(I). The same authors reported the activation of mainly the PERK pathway and increased ATF4 expression in cells with mutations in α1 and α2 as well as IRE1α pathway with a predominance in cells with mutations in α2, but there was no difference in the level of activated ATF6 [17]. In contrast, in our study, along with the increase in BiP expression, activation of both transcription factors ATF4 (effector of PERK pathway) and ATF6 in cells with BiP upregulation was found, while expression of spliced forms of XBP1 (effector of IRE1α pathway) was predominant in OI type III as confirmed by real-time PCR and polyacrylamide gel electrophoresis of (reverse transcriptase) RT-PCR product. It should be added that in our studies, the lack of increase in BiP expression in cells with G691C mutation was consistently associated with the lack of activation of UPR proteins, while in the case of some collagen and non-collagen mutations, activation of some UPR pathways was also observed in the absence of BiP [17,18]. It is possible that other factors or regulatory mechanisms are involved in the activation of the UPR during ER stress. In the osteoblasts of the mouse OI model with the substitution of glycine by cysteine (G610C), closely located to the one we studied (G691C) but in α2(I), no conventional UPR was detected also, only enhanced autophagy [15]. Moreover, as it turned out in our studies, the upregulation of genes of transcription factors ATF4 and ATF6 did not coincide with the increased amount of the protein, which means that their expression is regulated by post-transcriptional mechanisms and the expression of the genes themselves cannot be compared without determining the expression at the protein level.

Based on the obtained results, we can say that RE, if not completely eliminated, largely reduced ER stress caused by the accumulation of mutant collagen. This was evidenced by the decreased expression of chaperone proteins BiP and PDI as well as effectors of UPR branches along with the decreased level of intracellular collagen. Interestingly, in OI II 1 cells where we did not detect increased BiP expression and activation of UPR pathways, upregulation of pro-survival factors (BiP, ATF6 and XBP-1s), but not ATF4, was found in RE-treated cells. Activation of the PERK pathway leads to inhibition of global protein translation by inhibition of eIF2α except for ATF4, which in the active form can upregulate both the survival (autophagy) and the apoptotic (CHOP) pathway genes. In turn, the active transcription factors ATF6 a and XBP1s enhance the expression of chaperones, and also ATF6 of genes for proteins involved in ERAD [19,20,22]. While it is still unknown how activation of individual UPR pathways and their effectors directly affects collagen, one study found that forced XBP1s expression in cells with glycine substitution (G425S) in α1 (I) chain enhanced the folding/assembly and secretion of mutant type I collagen [38].

Since the cell response to ER stress caused by intracellular accumulation of mutant collagen is most often autophagy or, less frequently, ERAD, we investigated the activation of these two degradation systems in untreated and RE exposed OI cells. 

Autophagy, is a dynamic tightly regulated lysosomal pathway of degradation of intracellular components, including soluble proteins, aggregated proteins and damaged cell organelles. It is an evolutionarily conserved process, capable of responding to stress to limit cell damage. The autophagy process is regulated by several ATG core proteins, of which LC3 plays essential role in the formation and maturation of the autophagosome, [25,39]. Cytosolic form LC3-I is converted into an active membrane-bound form LC3-II during the formation of the autophagosomes, while the final degradation of the cargo takes place after fusion of autophagosome with lysosomes. It is strictly dependent on the p62, which apart from the ratio LC3-II/LC3I, is an important marker of effective autophagic flux [40]. Even though the LC3-II level was increasing in lethal untreated cells as compared to the aged-matched control, the ratio LC3-II/LC3-I remained unchanged, which along with the p62 increase indicated a lack of activation of autophagy. OI III cells showed an increase in the LC3-II/LC3-I ratio, but the p62 level remained unchanged, which also did not suggest an increase in autophagic activity. p62 with LC3 recognition sequence binds to LC3-II and after the formation of the autophagosome and its fusion with the lysosome, is degraded inside the autophagolysosome, that is why the decrease in p62 expression may indicate an active process of autophagy.

A markedly increased autophagic activity was observed in all OI cells after RE exposure, as shown by a dose-dependent increase in LC3-I to LC3-II conversion, along with accelerating p62 degradation. In addition, the stimulation of ATG5 mRNA and beclin 1 mRNA and protein, which initiate autophagosome formation, was demonstrated in RE-treated cells. Another evidence of the stimulating effect of RE on the degradation of mutant collagen with involvement of lysosomal pathway was the confirmation of the presence of collagen type I in the lysosomal fraction and the increase in its level in cells treated additionally with ammonium chloride, which raises the pH and thus inactivates lysosomes. The same results were obtained in the presence of chloroquine—another autophagy inhibitor (not shown). Moreover, the immunofluorescence microscopy studies have showed that collagen type I collocation increase in RE-treated OI cells with both the marker of autophagosome (LC3-II) and marker of lysosomes (LAMP2A). These results clearly indicate the RE-mediated degradation of mutated collagen type I in the autophagolysosomal process, although digestion of collagen by lysosomes regardless of autophagy cannot be ruled out. Omari et al. [41] showed that in addition to autophagy, accumulated in OI collagen type I can be digested in a noncanonical autophagy process. 

As reported earlier the proteasome may be involved in the removal of unfolded procollagen chains [27]. Misfolded proteins or unfolded procollagen chains are retranslocated from the ER to the cytosol for degradation by the 26S proteasome after modification with polyubiquitin chains. As expected, polyubiquitination of unfolded proα1(I) chains was demonstrated in OI III 2 with a C-propeptide mutation (G1448V). The increase in the amount of unfolded procollagen chains in untreated OI cells can be explained by decreased C-L and T-L proteasome activities. The greater accumulation of these chains in the presence of BR, which inhibited to a much greater extent all three activities (ChT-L, T-L and C-L) of the proteasome confirms the proteasome’s contribution to the removal of these chains. However, while it turned out that RE also decreased ChT-L, T-L and C-L activities, this decrease did not coincide with the accumulation of unfolded chains. On the contrary, there were lower levels of them than in untreated cells. At this stage of the study, it is difficult to explain the mechanism of action of RE in these cells, but it is very likely that in the case of inhibition of the proteasome activity, unfolded chains may be partially degraded in the process of RE-activated autophagy or by the proteasome, as the activity of the proteasome was only partially inhibited. It was also noted that, despite the reduction in proteasome activity by CQ, which inhibits autophagy, there was no additional accumulation of unfolded chains. On the other hand, another inhibitor of autophagy 3-MA that block autophagy at the initiation and maturation stages by acting on phosphoinositol 3 phosphate kinase (PI3K), caused a decrease in the level of unfolded chains compared to untreated OI cells, perhaps due to its stimulating effect on ChT-L activity. Since the two protein degradation systems (proteasome and autophagy) appear to be mechanically linked, it is suspected that when the proteasome is inhibited, autophagy may be activated to remove polyubiquitinated/unfolded protein aggregates and promote cell survival [42]. It is possible that a decrease in proteasome activity, also noted in other RE-treated OI cells used in this study (results not shown), triggers autophagy, protecting cells from the toxic long-term stress leading to cell apoptosis. The increase in the expression of pro-apoptotic proteins (Bax, CHOP and active caspase 3) in OI cells and their significant reduction or even normalization in the presence of RE, may unequivocally indicate that the accumulation of mutant type I collagen caused such stress and despite the activation of UPR (with the exception of OI II), the degradation processes mediated by autophagy and the proteasome were disturbed. The cell’s response to inhibited intracellular degradation processes could be a significant upregulation of extracellular MMP-1 and MMP-2 genes, lowered in the presence of RE, while the importance of these upregulation requires further study. Moreover, the observed increase in the level of extracellular type I collagen only in the presence of 100 µg/mL RE in OI III 1 can be explained by its strong inhibitory effect on the activity of MMP-2 and not by an increase in collagen secretion. Negative correlations between the activity of lysosomal enzymes and MMPs have been reported [43] and are worth studying further, but our research focuses on the intracellular degradation processes. 

It is also possible that, under the influence of RE, collagen folding is improved due to reduced over-modification of free procollagen chains as a RE-induced decrease in mRNA expression of one of the enzymes (β(1-O) galactosyltransferase (GLT25D1)) involved in these modifications was observed (data not shown).

Phytochemicals, due to their natural origin, low toxicity, as well as many valuable biological and pharmacological properties, are of wide interest among researchers as potential drugs with a high therapeutic and preventive potential for many diseases. *R. officinalis* is a polyphenol-rich source constituents such as luteolin and apigenin derivatives, caffeic acid derivative (rosmarinic acid) and other such as diterpenes (rosmanol isomers), detailed qualitative analysis of which was presented earlier [35]. 

In our previous studies, we have shown a stimulatory effect of rosemary and lemon balm extracts, RA as well as some flavonoids on collagen biosynthesis in OI type I [35,44] as well as normal human skin fibroblasts [45,46,47]. In these studies, the use of RA alone in a wide range of concentrations did not bring the expected effects, which may suggest the participation of other components of the rosemary extract in stimulating autophagy or its other effects. It has been reported by other authors that luteolin 7-*O*-glucoside (one of the identified RE components) protects against damage to the heart muscle induced by starvation by enhancing autophagy through inhibition of mechanistic target of rapamycin (mTOR) and extracellular signal-regulated kinase (ERK) signaling pathway [48]. It has also been shown that apigenin increases the expression of LC3-II, the formation of autophagolysosomal vacuoles and triggers autophagic flow in hepatocellular carcinoma cells [49]. It is widely accepted that a variety of plant extracts and dietary phytochemicals including resveratrol, curcumin, epigallocatechin-3-gallate, punicalagin, oleuropein, myricetin and rosmarinic, norhydroguaiaretic, and ferulic acids may stimulate autophagy [31,50]. These compounds remove protein aggregates, stimulate the antioxidant defense and ameliorate the ER stress, resulting in increased cell survival [31,32,33,34,50]. Pierzynowska et al. [51] reported on the removal of mutant huntingtin aggregates in the transfected HEK293 cells via genistein-induced autophagy, which may be the basis for the development of an effective therapy for this inherited neurodegenerative disease. Interestingly, like RE in OI cells, genistein showed significant inhibition of all protesome activities in the fibroblasts of patients with all types of mucopolysaccharidosis, which according to the authors, may lead to the stabilization of lysosomal enzymes and constitute a new approach in the treatment of this genetic disease [52]. 

A synergistic effect of several different compounds present in the rosemary extract may also be likely. While the biological properties (e.g., antioxidant) of polyphenols were previously related mainly to the structure of these compounds, now a more convincing explanation is modulating the activity and/or expression of key proteins for signaling cascades by interacting with them or modulating epigenetic regulation of gene expression. Therefore, it is believed that pleiotropic mechanisms and specific polyphenol-protein interactions are involved in their beneficial effects [53,54]. 

Finally, the limitations of this study should also be mentioned. Firstly, experiments were conducted on fibroblasts, which is related to the availability of biological material, and the disease mainly affects the skeletal system. On the other hand, collagen type I is a major component of skin and bone and, with a few exceptions, is similarly expressed in fibroblasts and osteoblasts. It is also worth noting that the activity of the proteasome may vary with age and even tissues or may be different in OI patients; therefore, more detailed studies are needed to understand the molecular mechanisms of RE-induced changes in proteasome activity. Although at this stage of our research we did not focus on explaining the consequences of proteasome inhibition by RE, the lack of accumulation of non-collagen proteins was shown. 

## 4. Materials and Methods

### 4.1. Chemicals

Dulbecco’s minimal essential medium (DMEM), fetal bovine serum (FBS) and phosphate-buffered saline (PBS) were obtained from Gibco (Thermo Fisher Scientific, Waltham, MA, USA); penicillin, streptomycin, and glutamine were purchased from Quality Biologicals Inc. (Gaithersburg, MD, USA). Radioimmunoprecipitation assay (RIPA) buffer, protease inhibitor cocktail (P8340), magnesium L-ascorbate, sodium dodecyl sulfate (SDS), dimethyl sulfoxide (DMSO), [3-(4,5-dimethylthiazol-2-yl)-2,5-diphenyltetrazolium bromide] (MTT), bovine serum albumin (BSA), pepsin, gelatin, NH_4_Cl, CQ, 3-MA, and MG132 were provided by Sigma-Aldrich Corp. (St. Louis, MO, USA). BR was a product of Selleck Chemicals (Houston, TX, USA). Proteasome substrates: N-Suc-LLVY-AMC (7-amido-4-methylcoumarin) was purchased from Sigma-Aldrich Corp. (St. Louis, MO, USA), Bz-VGR-AMC and Z-LLE-AMC were obtained from Enzo Life Sciences, Inc. (Farmingdale, NY, USA). Rosemary extract (RE) was prepared and characterized using LC-MS technique according to procedure described in our previous study [35]. RA was a product of BIOKOM (Warsaw, Poland).

### 4.2. Fibroblast Culture and Treatment

The study was performed on skin fibroblasts derived from two patients with severe OI type III and mutations in *COL1A1*: Gly901Ser (patient 1) and Gly1448Val (patient 2), and two patients with lethal OI type II and mutations in *COL1A1*: Gly691Cys (patient 1) and Gly352Ser (patient 2) as well as two age matched normal cells. The normal skin fibroblast line used as a control for OI type III was CRL-1474 obtained from American Type Culture Collection (Manassas, VA, USA), and as a control for OI type II the normal line was derived from the foreskin on the 7th day of life of the donor. Fibroblasts from skin biopsy of OI patients and healthy control were obtained after informed consent in accordance with the Declaration of Helsinki and was approved by Bioethical Committee of the Jagiellonian University in Kraków, Poland (KBET/108/B/2007). 

Fibroblasts were cultured in DMEM supplemented with 10% FBS, 2 mM glutamine, penicillin (50 U/mL) and streptomycin (50 µg/mL) at 37 °C in a humidified incubator in atmosphere containing 5% CO_2_. For experiments, fibroblasts were grown to 90% confluence and the cultured medium was replaced with fresh DMEM without serum, supplemented with 25 µg/mL of magnesium ascorbate, before addition of compounds. Compounds were stored at 4 °C as the concentrated stock solutions in DMSO and were diluted in medium prior to addition to cell cultures. Fibroblasts were treated with RE at the concentration of 1–100 µg/mL and RA at the concentrations of 1–100 µM for 24 h. In addition, cells were treated with autophagy inhibitors: 50 μM CQ, 50 mM NH_4_Cl and 5 mM 3-MA or proteasome inhibitors: 50 nM BR and 2.5 µM MG132, all of which were dissolved in DMSO and appropriately diluted before adding to cell cultures. In all experiments the concentration of DMSO did not exceed 0.05% (*v*/*v*).

### 4.3. MTT Test to Determine Viability of Treated Cells 

Fibroblasts (1 × 10^4^ cells per well) were treated with RE (1–200 µg/mL) for 24 h. Then cells were washed three times with PBS and MTT solution (0.5 mg/mL) was added for 4 h. After removing MTT solution 1 mL of 0.1 M HCl in absolute isopropanol was added to dissolve formazan crystals by thoroughly shaking on a plate shaker (BioSan, Riga, Latvia), and the absorbance at 570 nm was measured using a microplate reader (TECAN, Männedorf, Switzerland).

### 4.4. Quantitative Real-Time PCR

Total RNA was isolated from cultured cells using a Total RNA Mini Plus concentrator (A&A Biotechnology, Gdynia, Poland) and the concentration of RNA was determined using NanoDrop 2000 spectrophotometer (Thermo Fisher Scientific, Waltham, MA, USA). The equal amounts (1 µg) of total RNA were used to the synthesis of complementary DNA (cDNA) with the use of cDNA Synthesis Kit (Bioline, London, UK). Quantitative Real-time PCR (qRT-PCR) analysis was performed in the CFX96 Real-Time System thermal cycler (Bio-Rad, Hercules, CA, USA) using the SensiFAST™ SYBR kit (Bioline, London, UK). The expression of desired gene was normalized to the level of glyceraldehyde-3-phosphate dehydrogenase (GAPDH) and changes were calculated by the ΔΔCt method. The sequences of primers (Genomed, Warsaw, Poland) are shown in Supplementary Table S1. The qRT-PCR parameters were as follows: 30 s at 95 °C followed by 40 cycles: 10 s at 95 °C, 10 s at 60–62 °C and 20 s at 72 °C. The reaction products were verified by analysis of their melting curves.

### 4.5. XBP1 Splicing Analysis 

PCR mixture contained 1 µg of isolated RNA and primers (0.3 µM each): sense (5′-TCAG CTT TTA CGA GAG AAA ACT CAT GGC CT-3′) and antisense (5′-AGA ACA TGT GTG TCG TCC AAG TGT GTC GTC CAA GTG TG-3′) purchased in Genomed (Warsaw, Poland). Samples were incubated 30 min at 50 °C followed by 30 cycles at 94 °C, 60 °C, and 72 °C for 30 s each in the CFX96 Real-Time System thermal cycler (Bio-Rad, Hercules, CA, USA). Reaction products were analyzed by electrophoresis on 7% polyacrylamide gel and visualized with ethidium bromide.

### 4.6. Western Blot 

Cell layers were harvested using RIPA buffer (Sigma-Aldrich Corp., St. Louis, MO, USA) and protease inhibitor cocktail (P8340) (Sigma-Aldrich Corp., St. Louis, MO, USA). The conditioned media were collected and concentrated 10 times with Centrifugal Filter Units (10K) (Merck Millipore Ltd., Carrigtwohill, County Cork, Ireland). The concentration of total protein in cell lysates and media was measured using BCA Protein Assay Kit (Pierce, Rockford, IL, USA) and Coomassie Plus—The Better Bradford Assay Reagent (ThermoFisher Scientific, Rockford, IL, USA), respectively. For Western blot an equal amount of protein (20 µg) was loaded on polyacrylamide gel (7.5%, 10% or 12% depending on the molecular mass of protein). Proteins were transferred from gels onto Immobilon-P Transfer membranes (Merck Millipore Ltd., Tullagreen, Carrigtwohill, County Cork, Ireland), which were blocked with 5% (*w*/*v*) non-fat dried milk diluted in 50 mM Tris-HCl, pH 7.5, 500 mM NaCl, 0.05% (*v*/*v*) Tween 20 (TBS-T) for 1 h at room temperature. Then, membranes were washed with TBS-T and incubated overnight at 4 °C with solutions of the following primary monoclonal antibodies: mouse anti-collagen type I (1:1000; Santa Cruz Biotechnology Inc., Santa Cruz, CA, USA), rabbit anti-ATF4 (1:1000; Abcam, Cambridge, UK), rabbit anti-ATF6 (1.1000; Abcam, Cambridge, UK), rabbit Bax (1:1000; Cell Signaling Technology, Danvers, MA, USA), mouse Beclin-1 (1:1000; Cell Signaling Technology, Danvers, MA, USA), rabbit BiP (1:1000; Cell Signaling Technology, Danvers, MA, USA), mouse cleaved caspase-3 (1:1000; Santa Cruz Biotechnology Inc., Santa Cruz, CA, USA), mouse CHOP (1:1000, Cell Signaling Technology, Danvers, MA, USA), rabbit anti-procollagen I (1:1000; Abcam, Cambridge, UK), rabbit LC3 (1:1000, Cell Signaling Technology, Danvers, MA, USA), mouse anti-p62 (1:1000; Abcam, Cambridge, UK), mouse XBP-1s (1:1000; Cell Signaling Technology, Danvers, MA, USA), mouse poly ubiquitinylated proteins, multi ubiquitin chains (1:500; Biomol Int., Plymouth Meeting, PA, USA), and rabbit anti- β-actin (1:1000; Sigma-Aldrich Corp., St. Louis, MO, USA) as a loading control. In the next step the appropriate horseradish peroxidase conjugated secondary antibody: anti-mouse immunoglobulin G (IgG) (whole molecule) (1:2000; Sigma-Aldrich Corp., St. Louis, MO, USA), anti-rabbit antibodies (1:2000; Cell Signaling Technology, Danvers, MA, USA), anti-rabbit immunoglobulin G (IgG), Fc, HRP conjugate antibodies (1:2000; EMD Millipore Corp., Temecula, CA, USA) or anti-mouse IgG (whole molecule)—alkaline phosphatase antibody (1:2000; Sigma-Aldrich Corp., St. Louis, MO, USA) was added for 1 h with gentle shaking. After washing with TBS-T membranes were subjected to Westar Supernova Chemiluminescent Substrate for Western Blotting (Cyanagen, Bologna, Italy) and analyzed by densitometry (G:BOX, Syngene, Cambridge, UK). The intensity of analyzed proteins were normalized to β-actin which was a loading control. The data were expressed as a percentage of the normal sample taken as 100%. Determination of polyubiquitinated proteins was performed using Sigma Fast BCIP/NBT Alkaline Phosphatase Substrate (Sigma-Aldrich, St. Louis, MO, USA) by colorimetric detection (Gel Doc XR and Gel Documentation System; Molecular Imager Gel Doc XR, Bio-Rad Laboratories Inc., Hercules, CA, USA).

### 4.7. Immunofluorescence

Fibroblasts grown on cover-slips were fixed in 4% paraformaldehyde in PBS for 10 min at room temperature. After fixation, the cells were permeabilized in PBS containing 0.2% Triton-X100 for 5 min and blocked in 5 % normal donkey serum (Sigma-Aldrich Corp., St. Louis, MO, USA) at room temperature for 60 min to block non-specific reactions. Then cells were incubated with mouse monoclonal anti-collagen type I antibody (1:250, Santa Cruz Biotechnology Inc., Santa Cruz, CA, USA) and rabbit monoclonal anti-LC3B antibody (1:2000, Cell Signaling Technology, Danvers, MA, USA) or rabbit polyclonal anti-LAMP2A antibody (1:100, Abcam) for 60 min at room temperature. After incubation, the cells were washed three times with PBS and incubated in donkey anti-mouse IgG conjugated with Alexa Fluor 543 (1:200, Molecular Probes) or donkey anti-rabbit IgG conjugated with Alexa Fluor 488 (1:200, Molecular Probes) at room temperature for 1 h. Then, the cells were washed three times in PBS and stained with 4′,6′-diamidino-2-phenylindole (DAPI, Sigma-Aldrich Corp., St. Louis, MO, USA) for 10 min to indicate cell nuclei. The samples were washed twice with PBS and embedded in fluorescent medium (Medium Coverquick, Hygeco, OH, USA), dried overnight and stored in the dark until assessment. The immune labeled cells were analyzed using Nikon Digital Sight DS-Fi1 camera and a fluorescence microscope Nikon ECLIPSE Ti/C1 Plus, equipped with three filters DAPI (blue), FITC (green), and TRITC (red) (excitation wavelength/emission filter: 405/450 nm, 488/515 nm, 543/605 nm, respectively). No fluorescence signal was detected when cells were incubated with secondary antibodies alone (data not shown). At least five pictures of different areas of each treatment group were taken, independently analyzed and one representative image for each study group was presented.

### 4.8. Steady-State Analysis of Type I Collagen

Procollagens was extracted from cell lysates by precipitation overnight at 4 °C with ammonium sulfate (176 mg/mL). To obtain collagen, procollagen was subjected to digestion with pepsin (50 μg/mL) for 4 h at 4 °C. For electrophoretic analysis of migration of collagen chains, SDS-urea-PAGE (5% polyacrylamide gel) and silver staining were used.

### 4.9. Subcellular Fractionation 

Cells were suspended in buffer containing 40 mM KCl, 5 mM MgCl_2_, 2 mM EGTA, 10 mM HEPES, pH 7.5 for 30 min on ice. They were then homogenized by shearing 30 times through a 28.5-gauge needle and centrifuged at 1000× *g* for 10 min. The pellet was collected as the nuclear fraction, while the supernatant was subjected to centrifugation at 12,000× *g* for 10 min. The lysosome enriched pellet was washed using isotonic buffer (150 mM NaCl, 5 mM MgCl_2_, 2 mM EGTA, 10 mM HEPES pH 7.5) and dissolved in lysis buffer (1% Triton X-100, 150 mM NaCl, 50 mM Tris-HCl pH 7.5). The presence of collagen type I in this fraction was analyzed by Western blot. 

### 4.10. Determination of Proteasome Activities 

Cells were sonicated in lysis buffer containing 50 mM Tris-HCl (pH 7.5), 150 mM NaCl, 1 mM EDTA,1 mM EGTA, 0.5% Triton X, and centrifuged at 12,000× *g* for 15 min at 40 °C. The total protein concentration in supernatants was determined by the Bradford method in BioPhotometer (Eppendorf, Hamburg, Germany), using the Bio-Rad Protein Assay Dye Reagent Concentrate (Bio-Rad Laboratories Inc., Hercules, CA, USA) with BSA as a standard. The supernatants were diluted to concentration of 1.5 mg protein/mL in the lysis buffer. The reaction mixture (total volume of 50 μL) contained 30 μL of assay buffer (100 mM Tris/HCL, pH 7.5, 1 mM EDTA, 1 mM EGTA pH 7.5), 10 μL of cell lysate supernatant and 10 μL of fluorogenic peptide-AMC substrates: Suc-LLVY-AMC (Sigma Aldrich Corp., St. Louis, MO, USA) for chymotrypsin-like activity, Bz-VGR-AMC (Enzo Life Sciences, Inc., Farmingdale, NY, USA) for trypsin-like activity or Z-LLE-AMC (Enzo Life Sciences, Inc., Farmingdale, NY, USA) for caspase-like activity in a final concentration of 100 µM each [55]. The 96-well black plates (Corning Inc., Corning, NY, USA) were used and the assays were performed at 37 °C in FLUOStar OPTIMA (BMG Labtech Gmbh, Offenburg, Germany) over 30 min with one reading every 2 min, at 355 nm for excitation and 460 nm for emission. One unit of the proteasome activity was expressed as the amount of AMC released from the substrate per minute (pmol/min). The activity was calculated for the amount of total protein (U/mg). All assays were performed in triplicates.

### 4.11. Zymography for the Determination of MMP Activity

The conditioned media were collected and subjected non-reducing SDS–PAGE. The substrate for MMP was gelatin (1 mg/mL) (Sigma-Aldrich Corp., St. Louis, MO, USA). In order to remove SDS, gels were incubated in 2.5% Triton X-100 solution for 30 min at room temperature. In the next step, gels were incubated overnight in 50 mM Tris-HCl, pH 8.0, 5 mM CaCl_2_, 5 µM ZnCl_2_ and 0.02% NaN_3_) at 37 °C with gentle shaking. After that, they were stained with Commassie blue R-250 solution and destained until the appearance of white stripes on a dark blue background. Images of the zymograms were analyzed by densitometry (G:BOX, Syngene, Cambridge, UK).

### 4.12. Statistical Analysis

The results were statistically analyzed using the Statistica 12 software (StatSoft, Tulsa, OK, USA) and presented as the mean ± standard deviation (SD). Statistical differences were estimated by the use of one-way ANOVA followed by Tukey’s test and values of *p* < 0.05 were considered as significant. 

## 5. Conclusions

This study provides new insight into the effects of rosemary extract on OI fibroblasts with mutant collagen retention. The data presented here shows, for the first time, the pro-autophagy effect and the protective action of RE against the apoptosis of OI fibroblasts. Our findings could have important implications in OI treatment trials as RE removed accumulated mutant collagen and unfolded procollagen chains, improving cell homeostasis as indicated by decreased expression of UPR proteins. Reduction of proteasome activity by RE did not result in additional accumulation of non-collagen proteins. Although the exact mechanisms of the autophagy stimulatory and the proteasome and MMP (-1 and -2) inhibitory effects of RE require elucidation, the obtained results of our research are promising and worth continuing in order to understand the molecular pathways involved in the pathology of OI and beneficial effects of RE.

## Supplementary Materials

The following supporting information can be downloaded at: https://www.mdpi.com/article/10.3390/ijms231810341/s1.

## Abbreviations

3-MA	3-Methyladenine
4-PBA	4-Phenylbutyrate
ATF4	Activating transcription factor 4
ATF6	Activating transcription factor 6
BiP	Binding-Immunoglobulin Protein
BR	Bortezomib
C-L	caspase-like activity
Ch-L	chymotrypsin-like activity
CHOP	CCAAT enhancer binding protein
CQ	Chloroquine
DMEM	Dulbecco’s minimal essential medium
DMSO	Dimethyl sulfoxide
ECM	Extracellular matrix
eIF2	Eucaryotic translation initiation factor
ER	Endoplasmic reticulum
ERAD	Endoplasmic reticulum (ER)-associated degradation
FBS	Fetal bovine serum
IRE1	Inositol requiring enzyme 1
LAPM2A	Lysosomal-associated membrane protein 2A
LC3	Microtubule associated protein 1 Light Chain 3
MMPs	Matrix Metalloproteinases
NH_4_Cl	Ammonium chloride
OI	Osteogenesis imperfecta
P3H1	Prolyl 3-Hydroxylase 1
PBS	Phosphate-buffered saline
PERK	PKR-like endoplasmic reticulum kinase
PDI	Protein disulfide isomerase
RA	Rosmarinic acid
RE	Rosemary extract
RIPA	Radioimmunoprecipitation assay buffer
SDS	Sodium dodecyl sulfate
SDS-urea PAGE	SDS-urea polyacrylamide gel electrophoresis
SQSTM1/p62	Sequestosome-1
TGF-β	Transforming Growth Factor Beta
T-L	trypsin-like activity
UPR	Unfolded protein response
XBP-1s	X-Box binding protein 1

## References

[1] Forlino A., Cabral W.A., Barnes A.M., Marini J.C. (2011). New perspectives on osteogenesis imperfecta. Nat. Rev. Endocrinol..

[2] Marini J.C., Forlino A., Bächinger H.P., Bishop N.J., Byers P.H., Paepe A., Fassier F., Fratzl-Zelman N., Kozloff K.M., Krakow D. (2017). Osteogenesis imperfecta. Nat. Rev. Dis. Primers.

[3] Marom R., Rabenhorst B.M., Morello R. (2020). Osteogenesis imperfecta: An update on clinical features and therapies. Eur. J. Endocrinol..

[4] Gajko-Galicka A. (2002). Mutations in type I collagen genes resulting in osteogenesis imperfecta in humans. Acta Biochim. Pol..

[5] Lim J., Grafe I., Alexander S., Lee B. (2017). Genetic causes and mechanisms of Osteogenesis Imperfecta. Bone.

[6] Van Dijk F.S., Sillence D.O. (2014). Osteogenesis imperfecta: Clinical diagnosis, nomenclature and severity assessment. Am. J. Med. Genet. A.

[7] Willing M.C., Deschenes S.P., Scott D.A., Byers P.H., Slayton R.L., Pitts S.H., Arikat H., Roberts E.J. (1994). Osteogenesis imperfecta type I: Molecular heterogeneity for COL1A1 null alleles of type I collagen. Am. J. Hum. Genet..

[8] Jovanovic M., Guterman-Ram G., Marini J.C. (2022). Osteogenesis Imperfecta: Mechanisms and signaling pathways connecting classical and rare OI types. Endocr. Rev..

[9] Kang H., Aryal A.C.S., Marini J.C. (2017). Osteogenesis imperfecta: New genes reveal novel mechanisms in bone dysplasia. Transl. Res..

[10] Etich J., Leßmeier L., Rehberg M., Sill H., Zaucke F., Netzer C., Semler O. (2020). Osteogenesis imperfecta-pathophysiology and therapeutic options. Mol. Cell Pediatr..

[11] Ishikawa Y., Bächinger H.P. (2013). A molecular ensemble in the rER for procollagen maturation. Biochim. Biophys. Acta.

[12] Claeys L., Storoni S., Eekhoff M., Elting M., Wisse L., Pals G., Bravenboer N., Maugeri A., Micha D. (2021). Collagen transport and related pathways in Osteogenesis Imperfecta. Hum. Genet..

[13] Taga Y., Kusubata M., Ogawa-Goto K., Hattori S. (2013). Site-specific quantitative analysis of overglycosylation of collagen in osteogenesis imperfecta using hydrazide chemistry and SILAC. J. Proteome Res..

[14] Lisse T.S., Thiele F., Fuchs H., Hans W., Przemeck G.K., Abe K., Rathkolb B., Quintanilla-Martinez L., Hoelzlwimmer G., Helfrich M. (2008). ER stress-mediated apoptosis in a new mouse model of osteogenesis imperfecta. PLoS Genet..

[15] Mirigian L.S., Makareeva E., Mertz E.L., Omari S., Roberts-Pilgrim A.M., Oestreich A.K., Phillips C.L., Leikin S. (2016). Osteoblast malfunction caused by cell stress response to procollagen misfolding in α2(I)-G610C mouse model of Osteogenesis Imperfecta. J. Bone Miner. Res..

[16] Garibaldi N., Contento B.M., Babini G., Morini J., Siciliani S., Biggiogera M., Raspanti M., Marini J.C., Rossi A., Forlino A. (2021). Targeting cellular stress in vitro improves osteoblast homeostasis, matrix collagen content and mineralization in two murine models of osteogenesis imperfecta. Matrix Biol..

[17] Besio R., Iula G., Garibaldi N., Cipolla L., Sabbioneda S., Biggiogera M., Marini J.C., Rossi A., Forlino A. (2018). 4-PBA ameliorates cellular homeostasis in fibroblasts from osteogenesis imperfecta patients by enhancing autophagy and stimulating protein secretion. Biochim. Biophys. Acta Mol. Basis Dis..

[18] Besio R., Garibaldi N., Leoni L., Cipolla L., Sabbioneda S., Biggiogera M., Mottes M., Aglan M., Otaify G.A., Temtamy S.A. (2019). Cellular stress due to impairment of collagen prolyl hydroxylation complex is rescued by the chaperone 4-phenylbutyrate. Dis. Model Mech..

[19] Bateman J.F., Shoulders M.D., Lamandé S.R. (2022). Collagen misfolding mutations: The contribution of the unfolded protein response to the molecular pathology. Connect. Tissue Res..

[20] Boot-Handford R.P., Briggs M.D. (2010). The unfolded protein response and its relevance to connective tissue diseases. Cell Tissue Res..

[21] Sutkowska-Skolimowska J., Galicka A., Młynarczuk-Biały I., Biały Ł. (2021). ER stress in osteogenesis imperfecta (OI)-causative mutations and potential treatment. Advances in Biomedical Research—Cancer and Miscellaneous.

[22] Hetz C., Chevet E., Oakes S.A. (2015). Proteostasis control by the unfolded protein response. Nat. Cell Biol..

[23] Tsang K.Y., Chan D., Bateman J.F., Cheah K.S. (2010). In vivo cellular adaptation of ER stress: Survival strategies with double-edged consequences. J. Cell Sci..

[24] Ishida Y., Yamamoto A., Kitamura A., Lamandé S.R., Yoshimori T., Bateman J.F., Kubota H., Nagata K. (2009). Autophagic elimination of misfolded procollagen aggregates in the endoplasmic reticulum as a means of cell protection. Mol. Biol. Cell.

[25] Yang Z., Klionsky D.J. (2009). An overview of the molecular mechanism of autophagy. Curr. Top Microbiol. Immunol..

[26] Lamandé S.R., Chessler S.D., Golub S.B., Byers P.H., Chan D., Cole W.G., Sillence D.O., Bateman J.F. (1995). Endoplasmic reticulum-mediated quality control of type I collagen production by cells from osteogenesis imperfecta patients with mutations in the pro alpha 1 (I) chain carboxyl-terminal propeptide which impair subunit assembly. J. Biol. Chem..

[27] Fitzgerald J., Lamandé S.R., Bateman J.F. (1999). Proteasomal degradation of unassembled mutant type I collagen pro-alpha1(I) chains. J. Biol. Chem..

[28] Botor M., Fus-Kujawa A., Uroczynska M., Stepien K.L., Galicka A., Gawron K., Sieron A.L. (2021). Osteogenesis imperfecta: Current and prospective therapies. Biomolecules.

[29] Gioia R., Tonelli F., Ceppi I., Biggiogera M., Leikin S., Fisher S., Tenedini E., Yorgan T.A., Schinke T., Tian K. (2017). The chaperone activity of 4PBA ameliorates the skeletal phenotype of Chihuahua, a zebrafish model for dominant osteogenesis imperfecta. Hum. Mol. Genet..

[30] Takeyari S., Kubota T., Ohata Y., Fujiwara M., Kitaoka T., Taga Y., Mizuno K., Ozono K. (2021). 4-Phenylbutyric acid enhances the mineralization of osteogenesis imperfecta iPSC-derived osteoblasts. J. Biol. Chem..

[31] García-Aguilar A., Palomino O., Benito M., Guillén C. (2021). Dietary polyphenols in metabolic and neurodegenerative diseases: Molecular targets in autophagy and biological effects. Antioxidants.

[32] Fraga C.G., Croft K.D., Kennedy D.O., Tomás-Barberán F.A. (2019). The effects of polyphenols and other bioactives on human health. Food Funct..

[33] de Oliveira J.R., Camargo S.E.A., de Oliveira L.D. (2019). *Rosmarinus officinalis* L. (rosemary) as therapeutic and prophylactic agent. J. Biomed. Sci..

[34] González-Minero F.J., Bravo-Díaz L., Ayala-Gómez A. (2020). *Rosmarinus officinalis* L. (Rosemary): An ancient plant with uses in personal healthcare and cosmetics. Cosmetics.

[35] Sutkowska J., Hupert N., Gawron K., Strawa J.W., Tomczyk M., Forlino A., Galicka A. (2021). The stimulating effect of rosmarinic acid and extracts from rosemary and lemon balm on collagen type I biosynthesis in Osteogenesis Imperfecta type I skin fibroblasts. Pharmaceutics.

[36] Kirkness M.W., Lehmann K., Forde N.R. (2019). Mechanics and structural stability of the collagen triple helix. Curr. Opin. Chem. Biol..

[37] Makareeva E., Leikin S., Shapiro J.R., Byers P.H., Glorieux F.H., Sponseller P.D. (2014). Collagen structure, folding and function. Osteogenesis Imperfecta.

[38] DiChiara A.S., Doan N.D., Bikovtseva A.A., Rowley L., Butty V.L., Weis M.E., Eyre D.R., Lamandé S.R., Bateman J.F., Shoulders M.D. (2021). XBP1s-mediated endoplasmic reticulum proteostasis network enhancement can selectively improve folding and secretion of an osteogenesis imperfecta—causing collagen—I variant. bioRxiv.

[39] Mizushima N. (2018). A brief history of autophagy from cell biology to physiology and disease. Nat. Cell Biol..

[40] Sánchez-Martín P., Komatsu M. (2018). p62/SQSTM1-steering the cell through health and disease. J. Cell Sci..

[41] Omari S., Makareeva E., Roberts-Pilgrim A., Mirigian L., Jarnik M., Ott C., Lippincott-Schwartz J., Leikin S. (2018). Noncanonical autophagy at ER exit sites regulates procollagen turnover. Proc. Natl. Acad. Sci. USA.

[42] Wojcik S. (2013). Crosstalk between autophagy and proteasome protein degradation systems: Possible implications for cancer therapy. Folia Histochem. Cytobiol..

[43] Qin D., Ren R., Jia C., Lu Y., Yang Q., Chen L., Wu X., Zhu J., Guo Y., Yang P. (2018). Rapamycin protects skin fibroblasts from ultraviolet B-induced photoaging by suppressing the production of reactive oxygen species. Cell Physiol. Biochem..

[44] Galicka A., Nazaruk J. (2007). Stimulation of collagen biosynthesis by flavonoid glycosides in skin fibroblasts of osteogenesis imperfecta type I and the potential mechanism of their action. Int. J. Mol. Med..

[45] Nazaruk J., Galicka A. (2014). The influence of selected flavonoids from the leaves of Cirsium palustre (L.) Scop. on collagen expression in human skin fibroblasts. Phytother. Res..

[46] Matwiejczuk N., Galicka A., Zareba I., Brzóska M.M. (2020). The protective effect of rosmarinic acid against unfavorable influence of methylparaben and propylparaben on collagen in human skin fibroblasts. Nutrients.

[47] Galicka A., Sutkowska-Skolimowska J. (2021). The beneficial effect of rosmarinic acid on benzophenone-3-induced alterations in human skin fibroblasts. Int. J. Mol. Sci..

[48] Yao H., Zhou L., Tang L., Guan Y., Chen S., Zhang Y., Han X. (2017). Protective effects of luteolin-7-O-glucoside against starvation-induced injury through upregulation of autophagy in H9c2 cells. Biosci. Trends.

[49] Yang J., Pi C., Wang G. (2018). Inhibition of PI3K/Akt/mTOR pathway by apigenin induces apoptosis and autophagy in hepatocellular carcinoma cells. Biomed. Pharmacother..

[50] Yessenkyzy A., Saliev T., Zhanaliyeva M., Masoud A.R., Umbayev B., Sergazy S., Krivykh E., Gulyayev A., Nurgozhin T. (2020). Polyphenols as caloric-restriction mimetics and autophagy inducers in aging research. Nutrients.

[51] Pierzynowska K., Gaffke L., Hać A., Mantej J., Niedziałek N., Brokowska J., Węgrzyn G. (2018). Correction of Huntington’s disease phenotype by genistein-induced autophagy in the cellular model. Neuromolecular Med..

[52] Pierzynowska K., Gaffke L., Jankowska E., Rintz E., Witkowska J., Wieczerzak E., Podlacha M., Węgrzyn G. (2020). Proteasome composition and activity changes in cultured fibroblasts derived from mucopolysaccharidoses patients and their modulation by genistein. Front. Cell Dev. Biol..

[53] Hrelia S., Angeloni C. (2020). New mechanisms of action of natural antioxidants in health and disease. Antioxidants.

[54] Lacroix S., Klicic Badoux J., Scott-Boyer M.P., Parolo S., Matone A., Priami C., Morine M.J., Kaput J., Moco S. (2018). A computationally driven analysis of the polyphenol-protein interactome. Sci. Rep..

[55] Rodgers K.J., Dean R.T. (2003). Assessment of proteasome activity in cell lysates and tissue homogenates using peptide substrates. Int. J. Biochem. Cell Biol..

