# Supplementary material for: Rosemary Extract-Induced Autophagy and Decrease in Accumulation of Collagen Type I in Osteogenesis Imperfecta Skin Fibroblasts
Source: Int J Mol Sci. 2022 Sep 7;23(18):10341. doi: 10.3390/ijms231810341 (PMC9499644; doi:10.3390/ijms231810341)
Supplement: Supplementary file 1 [file ijms-23-10341-s001.zip › ijms-1890869-supplementary.pdf]

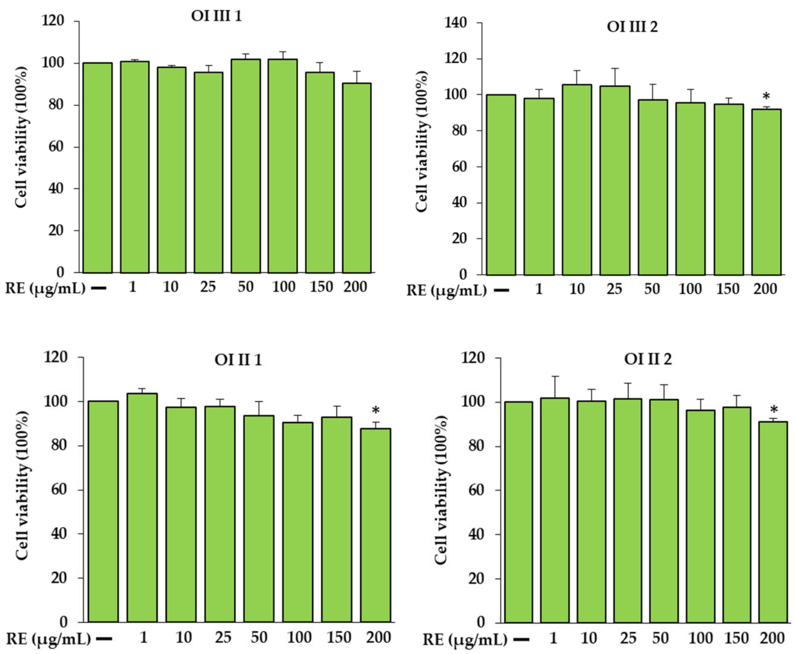

Figure S1. The effect of rosemary extract (RE) at concentrations of 1-200 μg/mL on the viability of OI types III and II fibroblasts of patients 1 and 2. Values represent the mean ± SD of three experiments done in duplicate; * $p < 0.05$, vs. untreated cells. The data are expressed as a percentage of the untreated sample taken as 100%.

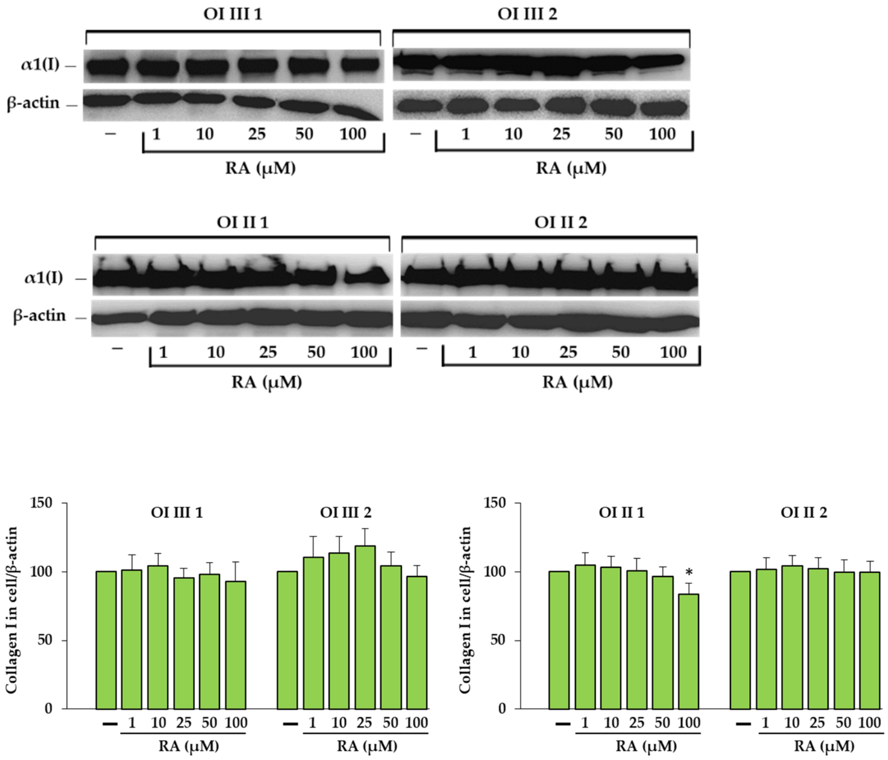

Figure S2. The effect of rosmarinic acid (RA) on the level of intracellular type I collagen in OI types III and II fibroblasts of patients 1 and 2; β-actin was used as cell protein loading control. The bars represent the results of the gel densitometry as the mean values from three independent experiments; * $p < 0.05$, vs. untreated cells. The data are expressed as a percentage of the untreated sample taken as 100%.

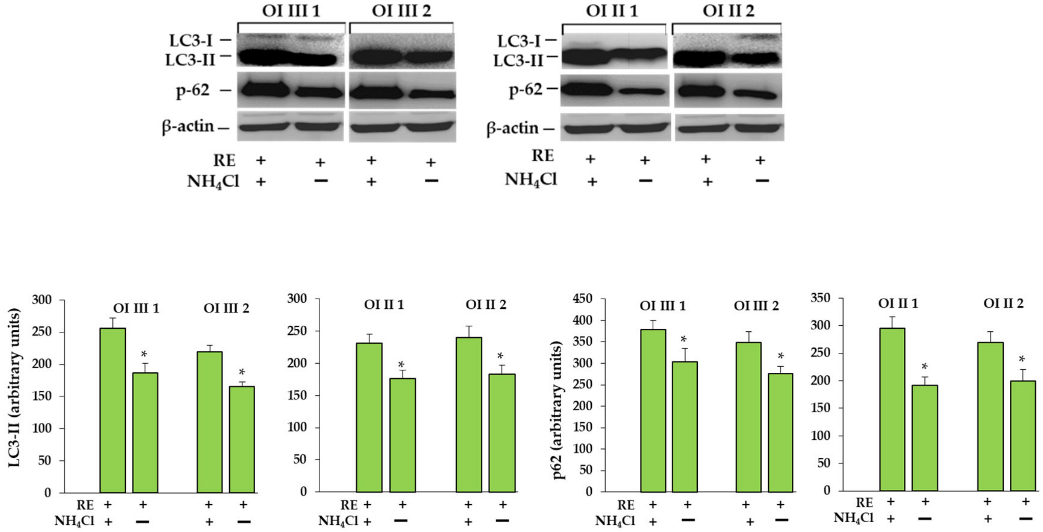

Figure S3. The increase in the level of LC3-II and p62 in the presence of inhibitor of autophagy (50 mM NH_4Cl) in OI cells treated with 50 µg/mL rosemary extract (RE); β-actin was used as cell protein loading control. The bars represent the results of the gel densitometry as the mean values from three independent experiments; * $p < 0.05$, OI cells treated with RE alone vs. OI cells treated with RE and NH_4Cl.

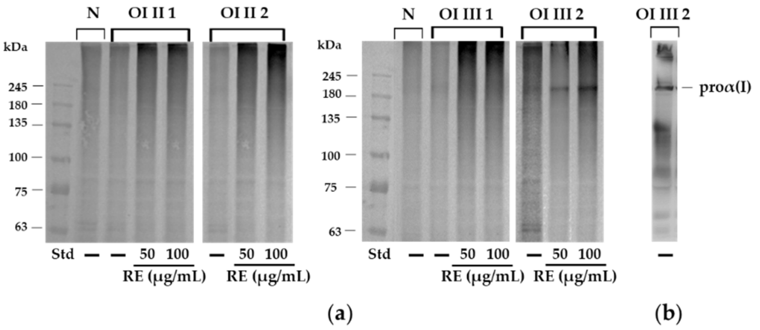

Figure S4. Western blot of polyubiquitynated proteins (**a**) and procollagen type I (**b**) followed SDS-PAGE under nonreducing conditions in normal (N), and untreated and treated with rosemary extract (RE) OI types III and II cells of patients 1 and 2. The arrows indicate the presence of polyubiquitynated high molecular proteins bands only in OI III 2 cells with the mutation in C-propeptide.

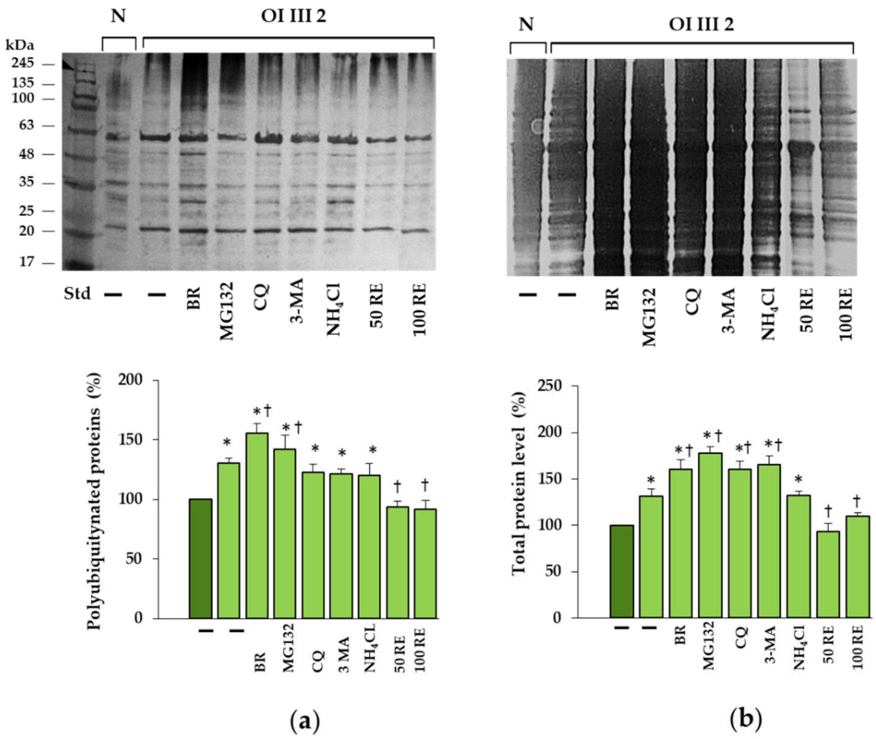

Figure S5. Western blot of polyubiquitinated proteins (**a**) and SDS-PAGE of total lysate proteins separated in reducing conditions and stained with silver salt (**b**) in normal (N) and OI type III cells of patient 2. OI cells were treated with 5 nM bortezomib (BR), 2.5 µM MG132, 50 µM chloroquine (CQ), 5 mM 3-methyladenine (3-MA), 50 mM NH_4Cl, and 50 and 100 µg/mL rosemary extract (RE). The bars represent the mean values from three independent experiments; * $p < 0.05$, OI cells vs. normal cells; † $p < 0.05$, OI treated cells vs. OI untreated cells. The data are expressed as a percentage of the normal sample taken as 100%; dark green and light green bars represent N and OI, respectively.

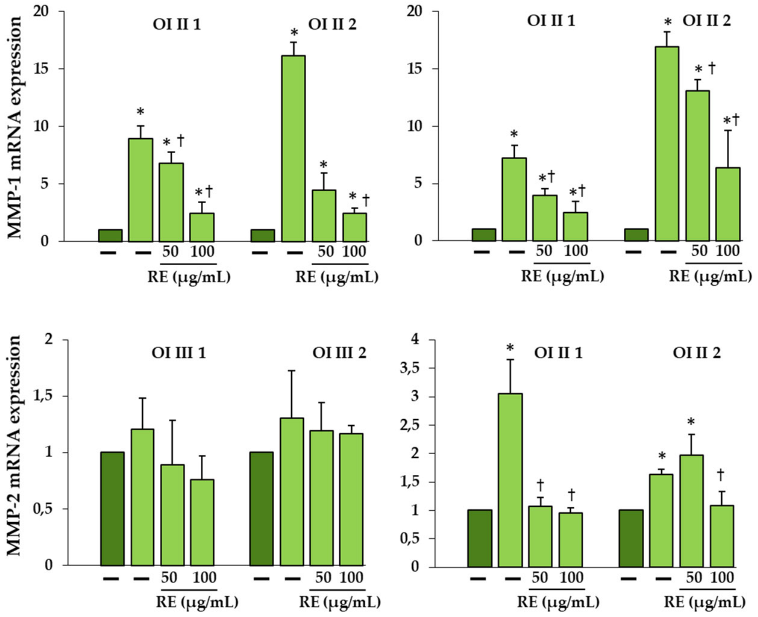

(**a**)

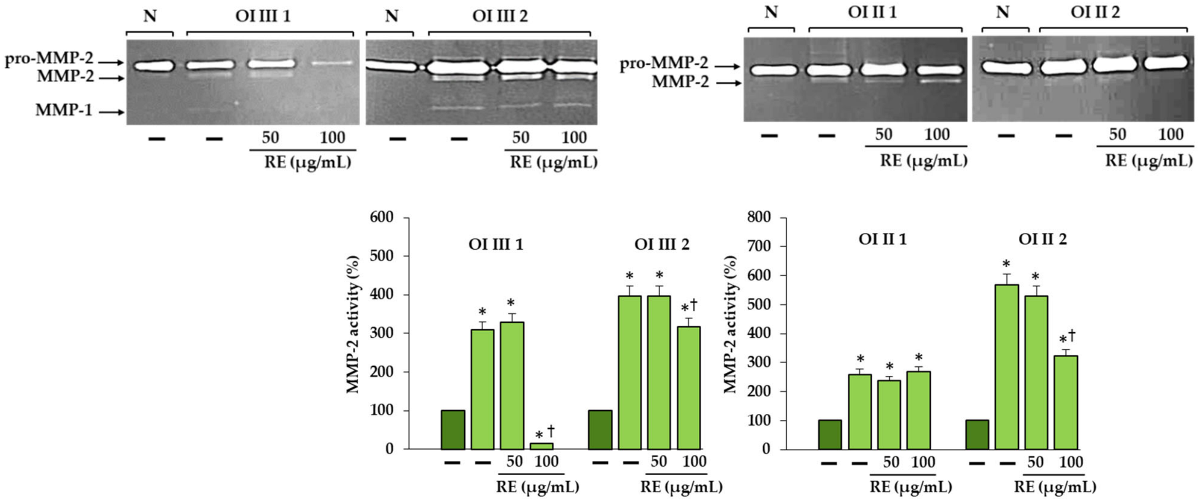

(**b**)

Figure S6. The influence of rosemary extract (RE) on the expression of matrix metalloproteinases (MMP-1 and MMP-2) mRNA (**a**) and MMP-2 activity (**b**) in OI types III and II cells of patients 1 and 2. The expression of MMP genes was assayed by real-time PCR, values represent the mean ± SD of three experiments (**a**). Representative gels of zymography (**b**); densitometry values represent the mean ± SD of three experiments. The data are expressed as a percentage of the normal (N) sample assumed as 100% (**b**); * $p < 0.05$, OI cells vs. normal cells; † $p < 0.05$, OI treated cells vs. OI untreated cells; dark green and light green bars represent N and OI, respectively.

Table S1. Sequences of primers used in the quantitative Real-Time PCR.

Gene		Primer sequence
ATG5	forward	5′-GGGAAGCAGAACCATACTATTTG-3′
	reverse	5′-AAATGTACTGTGATGTTCCAAGG-3′
ATF4	forward	5′-GTTCTCCAGCGACAAGGCTA-3′
	reverse	5′-ATCCTCCTTGCTGTTGTTGG-3′
ATF6	forward	5′ -TGAAGAGCAGGAAGGTGGTAG- 3′
	reverse	5′-AGGCAATGACAAAGAGTAGAAGG-3′
Bax	forward	5′-GGTGCCTCAGGATGCG-3′
	reverse	5′-GGAGTCTGTGTCCACG-3′
Beclin1	forward	5′-AGCTGCCGTTATACTGTTCTG-3′
	reverse	5′-ACTGCCTCCTGTGTCTTCAATCTT-3′
BiP	forward	5′-CGAGGAGGAGGACAAGAAGG-3′
	reverse	5′-CACCTTGAACGGCAAGAACT-3′
CHOP	forward	5′-GCGCATGAAGGAGAAAGAAC-3′
	reverse	5′- TCACAATTCGGTCAATCAGA -3′
PDI	forward	5′-GAGTTCTGCCACCGCTTCCT-3′
	reverse	5′-TGCTTGTCCCAGTCCTCCG-3′
MMP-1	forward	5′-CATTGATGGCATCCAAGCC-3′
	reverse	5′-GGCTGGACAGGATTTTGGG-3′
MMP-2	forward	5′-TGTGTCTTCCCCTTCACTTT-3′
	reverse	5′-GATCTGAGCGATGCCATCAA-3′
GAPDH	forward	5′-CTCTGCTCCTCCTGTTCGAC-3′
	reverse	5′-GCCCAATACGACCAAATCC-3′
